# Improving the Accuracy of Continuous Blood Glucose Measurement Using Personalized Calibration and Machine Learning

**DOI:** 10.3390/diagnostics13152514

**Published:** 2023-07-27

**Authors:** Ranjita Kumari, Pradeep Kumar Anand, Jitae Shin

**Affiliations:** 1 Department of Electrical and Computer Engineering, Sungkyunkwan University, Gyeonggi, Suwon 16419, Republic of Korea; ranjita@skku.edu; 2 Clinical Research Group, Samsung Healthcare, Gangdong-gu, Seoul 05340, Republic of Korea; pradeep@skku.edu

**Keywords:** diabetes, continuous blood glucose, personalized calibration, multilayer perceptron, machine learning

## Abstract

Despite tremendous developments in continuous blood glucose measurement (CBGM) sensors, they are still not accurate for all patients with diabetes. As glucose concentration in the blood is <1% of the total blood volume, it is challenging to accurately measure glucose levels in the interstitial fluid using CBGM sensors due to within-patient and between-patient variations. To address this issue, we developed a novel data-driven approach to accurately predict CBGM values using personalized calibration and machine learning. First, we scientifically divided measured blood glucose into smaller groups, namely, hypoglycemia (<80 mg/dL), nondiabetic (81–115 mg/dL), prediabetes (116–150 mg/dL), diabetes (151–181 mg/dL), severe diabetes (181–250 mg/dL), and critical diabetes (>250 mg/dL). Second, we separately trained each group using different machine learning models based on patients’ personalized parameters, such as physical activity, posture, heart rate, breath rate, skin temperature, and food intake. Lastly, we used multilayer perceptron (MLP) for the D1NAMO dataset (training to test ratio: 70:30) and grid search for hyperparameter optimization to predict accurate blood glucose concentrations. We successfully applied our proposed approach in nine patients with type 1 diabetes and observed that the mean absolute relative difference (MARD) decreased from 17.8% to 8.3%.

## 1. Introduction

**Figure 1:**
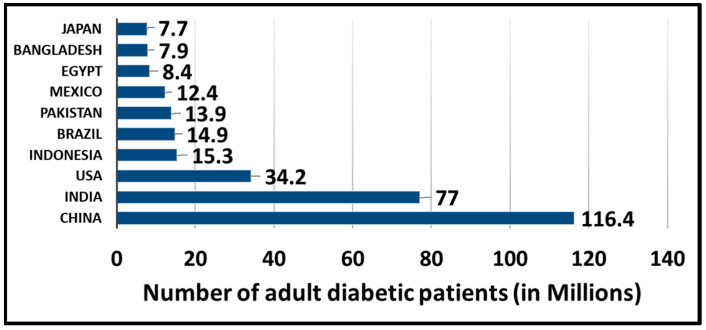
Countries with the highest number of patients with diabetes.

According to the International Diabetes Federation, in 2021, approximately 537 million adults (aged 20–79 years) were living with diabetes worldwide. This statistic indicates that approximately 1 adult per 10 adults in this age group is diabetic [1]. This number is increasing every year, and it is predicted that more than 783 million individuals will have diabetes by 2045 [1]. As of 2021, there were 116.4 million diabetic patients in China, 77.0 million in India, and 34.2 million in the United States of America (USA) [2] (Figure 1), and these numbers are constantly increasing.

### 1.1. Causes and Types of Diabetes

Diabetes mainly occurs due to an insulin disorder. For example, ineffective production of insulin by pancreatic beta cells can lead to diabetes, and this may occur from birth [3]. This type of diabetes is known as type 1 diabetes. Moreover, ineffective use of insulin inside the body leads to type 2 diabetes [3]. The symptoms of type 1 diabetes include excess urination, thrush, hunger, weight loss, and vision change. Patients with this type of diabetes need to monitor their blood glucose levels regularly and self-administer insulin in the form of an injection. The main cause of type 1 diabetes is high blood glucose levels, which lead to insulin resistance and ineffective production of insulin. According to the World Health Organization, approximately 10% of patients have type 1 diabetes and 90% have type 2 diabetes [3]. Both type 1 and type 2 diabetic patients require diagnostic and regular monitoring to manage their disease. Thus, the market for diabetes diagnostic products is large, with a global value of USD 28.1 billion in 2020 reported by StrategyR, a global industry analyst [4].

### 1.2. Biological Measurements of Blood Glucose

Glucose, as a biomarker for diabetes, can be measured in saliva, tears, sweat, urine, and blood [5]. The glucose concentration in these biological mediums ranges from 0.144 to 540 mg/dL. The glucose concentration is much lower in the saliva, tears, sweat, and urine (0.144–99 mg/dL) than in the blood (36–540 mg/dL) [5]. Detection of lower glucose concentrations requires highly accurate sensing technology; thus, the first four mediums are of limited clinical use. Blood has the highest glucose concentration and is therefore considered the best biological medium for glucose level measurement. To measure blood glucose concentration, a finger-prick test is often used. However, this is an invasive measurement method. Repeated pricking to obtain blood samples is painful. Instead of repeated finger pricks, a better approach for severely ill patients with type 1 diabetes involves placing a sensor beneath the skin once every few days (usually 7–14 days as per the manufacturer’s recommendations) to access the interstitial fluid and measure blood glucose concentrations continuously. This minimally invasive method for blood glucose measurement is called continuous blood glucose measurement (CBGM). The CBGM system provides real-time glucose readings, allowing individuals to understand their glucose trends, make informed decisions regarding their diabetes management, and adjust their doses of insulin or other medications accordingly. CBGM technology has significantly improved diabetes care by providing valuable insights into glucose fluctuations and helping severally ill patients with diabetes achieve better glycemic control [6].

### 1.3. Accuracy Assessment and Food and Drug Administration (FDA) Regulation

The mean absolute relative difference (MARD) and the Clarke error grid analysis (CEGA) plot are used to assess the accuracy of blood glucose measuring devices. For the MARD, the absolute percentage of errors for all measured blood glucose values is calculated in comparison with reference values. The MARD is an average value of all absolute percentage errors [7]. In 1987, Dr. William L. Clarke established a method for determining the accuracy of blood glucose devices [8]. In his established method, each measured blood glucose value is plotted with respective reference values. Then, based on the clinical criticality, the plot is divided into five zones, namely, A, B, C, D, and E. Zone A has error values <20% with respect to the reference. Zones B, C, D, and E can have different and higher error ranges based on the benign condition of patients with diabetes, deviation within hypoglycemia/hyperglycemia, failure to detect hypoglycemia/hyperglycemia, and confusing hypoglycemia for hyperglycemia, or vice versa, respectively [8].

According to the FDA, for adjunctive use, a blood glucose measurement device must have a MARD of ≤20 mg/dL for sensor glucose values <100 mg/dL and ≤20% for sensor glucose values ≥100 mg/dL for adjunctive use [9,10]. However, for nonadjunctive use (i.e., a blood glucose measurement device that can make insulin dosing decisions without confirming with a fingerstick test), the blood glucose measurement device must have a MARD of ≤10 mg/dL for sensor glucose values <100 mg/dL and ≤10% for sensor glucose values ≥100 mg/dL [9,10].

### 1.4. Existing Invasive, Minimally Invasive, and Noninvasive Methods for Measuring Blood Glucose

Several highly accurate and FDA-approved invasive blood glucose measurement devices have been developed in past decades. These include the Nova StatStrip Glucose Hospital Meter System, Abbott Precision Xceed Pro System, Nova Max Plus Glucose Meter, Roche Accu-Chek Aviva Plus System, Bayer Contour Next EZ System, and OneTouch Verio IQ System with MARDs of 5%, 5.5%, 6.1%, 5.1%, 5.8%, and 5.4%, respectively [11,12,13]. These devices are categorized as self-blood glucose measurement (SBGM) devices because they are used by individuals with diabetes to monitor their blood sugar levels and make informed decisions regarding diabetes management. Although these invasive devices are highly accurate, they require patients to prick their fingers for every measurement.

To relieve patients from frequent pricking, minimally invasive CBGM devices have been developed. CBGM sensors continuously measure glucose levels in the interstitial fluid over a period of time. They typically comprise a small, flexible probe that is inserted into the skin and connected to a transmitter or receiver that sends glucose data to a monitoring device, such as a smartphone or insulin pump. Some FDA-approved CBGM devices are G6 (9% MARD) from Dexcom [14], FreeStyle Libre 2 (9.3% MARD) and Libre 3 (9.7% MARD) from Abbott [15], Guardian Sensor 3 (9.4% MARD) and Guardian Connect (10.2% MARD) from Medtronic [16], and Eversense (9.6% MARD) from Senseonics [17]. CBGM is a notable advancement in blood glucose monitoring, with a tradeoff between one-time pricking and accuracy. However, the accuracy of CBGM needs to be improved to allow all patients with diabetes to administer insulin accurately and prevent hyperglycemia and hypoglycemia. Recently, researchers used machine learning techniques to improve CBGM. In a recent study, a stacked long short-term memory (LSTM)-based deep recurrent neural network model was used to predict blood glucose levels [18]. For the OhioT1DM dataset, average RMSEs of 6.45 and 17.24 mg/dL were achieved for 30- and 60-min prediction horizons, respectively. In a similar study, an LSTM-based neural network was designed to predict glucose levels for up to 60 min using continuous glucose measurements and the Tidepool Big Data Donation Dataset [19]. In that study, the RMSEs were 19.8 ± 3.2 and 33.2 ± 5.4 mg/dL for 30- and 60-min prediction horizons, respectively. These approaches can be used to predict future events of hyperglycemia and hypoglycemia, giving them a different purpose than our proposed approach.

Another study titled “Exploring noninvasive features for continuous glucose monitoring” was performed at the University of Memphis [20]. In that study, the researcher summarized different minimally invasive (glucose oxidase needle) and noninvasive (electrical impedance spectroscopy; metabolic heat confirmation; and GlucoTrack using a combination of ultrasonic, electromagnetic, and thermal technologies) sensors used for accurate blood glucose measurement using different machine learning models (linear regression, support vector regression (SVR), k-nearest regression, decision tree (DT) regression, bagging trees regressor, random forest (RF) regressor, Gaussian process regression, and multilayer perceptron (MLP)) [20]. However, that study did not divide the entire blood glucose range into smaller clusters or groups. Noninvasive blood glucose monitors (NIBGMs) show extremely large errors in the measurement of low glucose concentrations (<1%) in the blood using a noninvasive sensor because of within- and between-patient variations. Therefore, it is important to scientifically divide the entire blood measurement range into smaller clusters or groups and train machine learning models for each cluster separately to accurately predict a noninvasive value.

Several noninvasive sensors have also been developed over the last few decades. Some of these noninvasive sensors (and their accuracies) are based on infrared spectroscopy (85% in zone A), impedance spectroscopy (56% in zone A), diffuse reflectance spectroscopy (87.5% in zone A), Raman spectroscopy (86.7% in zone A), optical coherence tomography (11.5% MARD, 83% in zone A), photoacoustic spectroscopy (11.8% MARD, 82.7% in zone A), and a combination of these technologies (8.3% MARD, 90% in zone A) [21]. Using these noninvasive sensing technologies, a few successful NIBGMs have been developed, including Integrity Applications’ GlucoTrack (23.4% MARD, 57% in zone A) [22,23] and CNOGA’s CoG (17.1% MARD, 86.2% in zone A) [24]. Although there have been many developments in the field of noninvasive sensing technology, no NIBGM device has yet received FDA approval.

### 1.5. Role of Machine Learning in Blood Glucose Measurement

Recently, several attempts have been made to improve the accuracy of minimal invasive monitors using machine learning techniques. Some of the supervised machine learning models suitable for CBGM are described in this section.

SVR is a type of regression analysis that uses support vector machines to identify a hyperplane that minimizes the error between the predicted and actual values. It is commonly used for regression problems with a high degree of complexity, and it works well for data that are not linearly separable [25].

The k-nearest neighbor (KNN) algorithm is a nonparametric algorithm that makes predictions by identifying the k-nearest neighbors to a new observation and using their known outputs to estimate the output of the new observation. KNN works well for datasets with complex decision boundaries and is often used for classification problems [26].

DT is an algorithm that makes predictions by recursively partitioning the data into subsets based on the most informative features. The resulting tree structure can be used for both classification and regression problems and is often used for problems with categorical or discrete input features [27].

RF is a machine learning technique that combines the power of multiple DTs to make accurate predictions. Each DT in the RF is built using a different subset of the training data and features, adding an element of randomness to the process [28].

Adaptive boost (AdaBoost) is an ensemble learning algorithm that combines weak learners to create strong learners. Each weak learner is trained on a subset of the data and given a weight based on its performance. The final prediction is made by weighing the output of each weak learner based on its accuracy [29].

MLP is a neural network composed of multiple layers of interconnected nodes, each performing a simple computation on the input data. MLP is commonly used for problems with complex, nonlinear relationships between input and output variables [30].

Overall, each of these machine learning techniques has its own strengths and limitations, and the optimal algorithm depends on the sensor type and within-/between-patient variations in the dataset being analyzed.

### 1.6. Our Motivation and Contributions

The accuracy of CBGM depends on several factors other than sensor accuracy and the algorithm. These factors include within-patient and between-patient variations, which play an important role in the accuracy of CBGM. Within-patient variations include variations in food intake, physical activities, stress, skin temperature, and adipose tissue thickness. Between-patient variations include variations in insulin production by the pancreas and sensor location. Hence, we developed an algorithm using machine learning techniques and knowledge-based clustering that is adaptive and can intelligently learn errors in minimal invasive sensing owing to patient-to-patient variations. Once the predictive error model is developed during the calibration period, based on sensing technology and within- and between-patient variations, it accurately predicts CBGM values.

In the present study, we focused on explaining the proposed algorithm that defined the knowledge-based clusters based on the insulin production levels of patients with type 1 diabetes (“cluster 0: hypoglycemia” for blood glucose levels of <80 mg/dL; “cluster 1: nondiabetic” for blood glucose levels of 81–115 mg/dL; “cluster 2: prediabetes” for blood glucose levels of 116–150 mg/dL; “cluster 3: diabetes” for blood glucose levels of 151–181 mg/dL; “cluster 4: severe diabetes” for blood glucose levels of 181–250 mg/dL; and “cluster 5: critical diabetes” for blood glucose levels of >250 mg/dL); identifying within- and between-patient sources of variation for blood glucose, such as physical activity, peak acceleration, posture, heart/breath rate (representing stress), skin temperature, and food intake; determining suitable machine learning models (SVR, KNN, DT, RF, AdaBoost, and MLP) based on sensors used to measure the blood glucose according to the features identified in a patient and smaller clusters; and accurately predicting and providing blood glucose values. The proposed algorithm can be applied to any sensor, including those used for CBGM (minimally invasive monitor) and noninvasive blood glucose measurement.

We trained and tested CBGM using the D1NAMO dataset, which comprised data collected from nine patients with type 1 diabetes under real-life conditions by the University of Applied Sciences and Arts, Western Switzerland, used our proposed approach to create smaller clusters, train different machine learning models, and identify the suitable machine learning model for this dataset. Consequently, our proposed approach showed that the MARD of the predicted blood glucose values was significantly reduced compared with that of the measured blood glucose values. All data points for the predicted values fell within zone A of the CEGA plot.

The rest of this paper is organized as follows. Section 2 covers the multimodel machine learning approach for CBGM and its application to the D1NAMO dataset. Section 3 presents detailed results for MLP-based CBGM grid search, hyperparameter optimization, MARD values, RMSE values, sum of square error (SSE) plots, CEGA plots, and error plots for the predicted blood glucose values. The MLP-based CBGM results are discussed in Section 4. Finally, Section 5 concludes this paper.

## 2. Materials and Methods

Here, we elaborate on the advancement of our proposed approach for the accurate prediction of blood glucose values, as described below.

### 2.1. Multimodel Machine Learning Approach for CBGM

**Figure 2:**
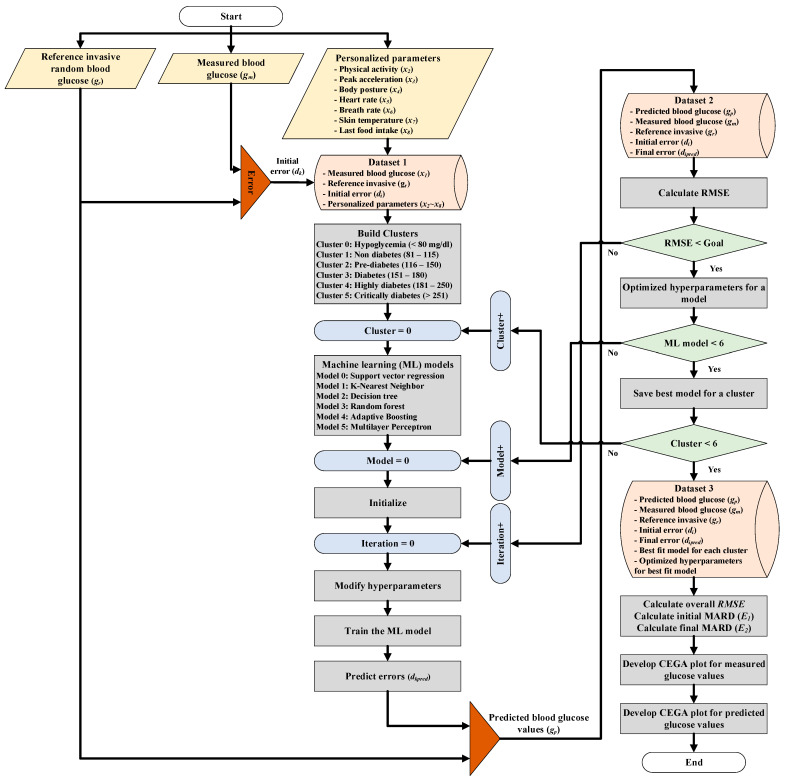
CBGM multimodel machine learning architecture diagram for accurate blood glucose concentration prediction using personalized parameters (physical activity, peak acceleration, posture, heart rate, breath rate, skin temperature, and food intake).

The architecture of our proposed multimodel CBGM is illustrated in Figure 2. Table 1 lists all parameters used in this paper, along with their symbols and definitions. First, minimally invasive measured blood glucose $\left(g_{m}\right)$ was compared along with reference invasive values $\left(g_{r}\right)$ during calibration, as shown in Figure 2. The paired readings $\left(g_{m},g_{r}\right)$ for each patient were collected along the patient’s personalized parameters. These personalized parameters included physical activity $\left(x_{2}\right)$, peak acceleration $\left(x_{3}\right)$, posture $\left(x_{4}\right)$, heart rate $\left(x_{5}\right)$, breath rate $\left(x_{6}\right)$, skin temperature $\left(x_{7}\right)$, and food intake $\left(x_{8}\right)$, as shown in Figure 2. Several paired readings with personalized parameters were taken for an individual and a group of patients with diabetes. Our proposed machine learning software analyzed the data and calculated the initial errors $\left(d_{k}\right)$.

The paired data, accompanying initial errors, and respective personalized parameters were termed dataset 1, as listed in Figure 2. We used the same cluster definition as published by the PGMS article, which was based on the stages of diabetes, as insulin produced by beta cells in the pancreas behaves differently at different blood glucose levels [31].

These clusters were named “cluster 0: hypoglycemia”, for blood glucose levels of <80 mg/dL; “cluster 1: nondiabetes”, for blood glucose levels of 81–115 mg/dL; “cluster 2: prediabetes”, for blood glucose levels of 116–150 mg/dL; “cluster 3: diabetes”, for blood glucose levels of 151–181 mg/dL; “cluster 4: severe diabetes,” for blood glucose levels of 181–250 mg/dL; and “cluster 5: critical diabetes”, for blood glucose levels of >250 mg/dL, as shown in Figure 2. These clusters were made to divide the measurement range of blood glucose into smaller groups, thereby avoiding large measurement errors and building better predictive models. Another reason for making these clusters was that the insulin produced by pancreatic beta cells behaves differently based on the blood glucose range in patients with diabetes. Hence, the pattern of the initial error in each group was different and strongly correlated with the blood glucose range and stage of diabetes. Eventually, these groups and patterns helped to develop robust machine learning models for the accurate prediction of blood glucose concentrations.

Next, we built different machine learning models for clusters 0–5. As shown in Figure 2, we used SVR, KNN, DT, RF, AdaBoost, and MLP machine learning techniques, as they all are supervised learning algorithms and can be used to solve regression-based problems that involve complex, nonlinear relationships between input and output variables. The within- and between-patient variations in diabetic patients are highly complex and nonlinear; hence, these machine learning models were considered suitable for our research. The software trains each of these machine learning models to calculate blood glucose values accurately. Once all models are trained, our proposed software predicts the error $\left(d_{kpred}\right)$ in the initially measured blood glucose values. Based on the predicted error, the software accurately predicts the blood glucose values $\left(g_{p}\right),$ as shown in Figure 2. We referred to dataset 2, which includes measured and predicted blood glucose values along with reference values with initial and final errors. Using dataset 2, we calculated the RMSE for each model and its corresponding set of hyperparameters. The model that gave the smallest RMSE value and met the preset accuracy goal was chosen along with a set of optimized hyperparameters for that cluster. This process was repeated for all clusters and their corresponding machine learning models until the optimal machine learning model for the target sensor and dataset was found, and it was named dataset 3.

Once the optimized models were trained and tested for each cluster, the software calculated the overall initial and final MARD for the measured and predicted values using dataset 3, as described in Figure 2. In addition, we generated a CEGA plot for the measured and predicted values to analyze the improvement in the accuracy of blood glucose measurement. Low values for the overall RMSE, low values for the MARD, and all data in zone A of the CEGA plot proved that the software accurately predicted blood glucose values.

The software was written in Python version 3.11 (the most up-to-date version at the time of writing). Moreover, it uses the Scikit-learn, Pandas, and Matlab libraries. The code successfully implemented the approach described in Figure 2. We used 70% and 30% of the data for training and testing the model, respectively.

### 2.2. Experimental Dataset

We referred to the D1NAMO dataset collected by the University of Applied Sciences and Arts Western Switzerland, Sterre, Switzerland [32]. The D1NAMO dataset is available publicly for scientific research in the field of diabetes. The data in this dataset were collected from 20 healthy controls and 9 patients with type 1 diabetes in real-life conditions. We downloaded 64 GB of data from D1NAMO. The data in the D1NAMO dataset are divided into diabetic and healthy subsets. We were interested only in the diabetic subset. Each subject in the diabetic subset was clearly labeled and further divided based on device data, glucose values, food intake, and insulin information. The device data contained several files with an enormous amount of information. However, we were interested in files named “Summary.csv.” This file contained information on 34 parameters that were recorded based on time for several days for each patient using the Zephyr BioHarness 3 wearable device [33]. The 34 parameters included heart rate, breath rate, skin temperature, posture, activity, peak acceleration, battery voltage, breath rate amplitude, breath rate noise, breath rate confidence, electrocardiogram (ECG) amplitude, ECG noise, heart rate confidence, heart rate variability (HRV), system confidence, GSR, ROG state, ROG time, vertical minimum, lateral peak, sagittal minimum, sagittal peak, device temperature, status information, link quality, RSSI, transmission power, core temperature, auxiliary ADC1, auxiliary ADC2, and auxiliary ADC3. Other CSV files in the D1NAMO dataset contained blood glucose values, food intake data, and insulin intake data.

We preprocessed the data based on the requirements of CBGM. We extracted relevant personalized and blood glucose data. The extracted personalized data included data on heart rate, breath rate, skin temperature, posture, activity, peak acceleration, HRV, and device temperature. After analyzing the data, we found that the HRV and skin temperature data contained many errors (default values), possibly due to the nonfunctioning of the sensor. Thus, we did not use these two parameters. We considered that the device temperature closely represented the skin temperature and hence included it in the extracted personalized data. The D1NAMO dataset contains measured blood glucose values from two different devices for patients with diabetes. These devices are minimally invasive CBGM and invasive SBGM. The CBGM device was Medtronic iPro2 Professional [34], which uses a glucose sensor based on glucose oxidase chemistry. Invasive measurement was performed by patients who owned a highly accurate self-monitoring device (for example, SBGM from Abbott or Roche or Bayer). The CBGM measurements were performed every 5 min, whereas manual measurement was performed once before a meal and 2 h after a meal. The D1NAMO dataset has very few manual glucose measurements as it is an invasive method, but it is highly accurate.

Hence, our final extracted paired blood glucose data comprised 166 readings from 9 patients with diabetes. As these readings were time-classified, we merged paired glucose readings with extracted personalized data and prepared input experimental data for our proposed approach. The D1NAMO food data were categorized as balanced or unbalanced and low, medium, or good quality based on the food type and calories. We also mapped the food information in our proposed-approach experimental data to model the real-life conditions.

### 2.3. Initial Results of Multimodel CBGM Based on the Experimental Dataset

We applied our proposed approach to the experimental dataset consisting of 166 readings with 8 input features. The following machine learning models were trained and tested:

1. SVR;
2. KNN;
3. DT;
4. RF;
5. AdaBoost;
6. MLP.

The initial test results are summarized in Table 2, which were used to screen the best-performing machine learning model. From Table 2, it is clear that MLP outperforms SVR, KNN, DT, RF, and AdaBoost for the experimental dataset obtained from D1NAMO. MLP achieved a MARD of 14.4% compared with 24.9% for SVR, 23.9% for KNN, 17.4% for DT, 16.6% for RF, and 15.6% for AdaBoost. MLP also had the best CEGA plot zonal result compared with all other models. A neural network performs well with a higher number of input features. Thus, MLP outperforms the experimental dataset obtained from D1NAMO, which has eight input features. Therefore, we decided to use the MLP machine learning model for further research.

### 2.4. MLP-Based CBGM

**Figure 3:**
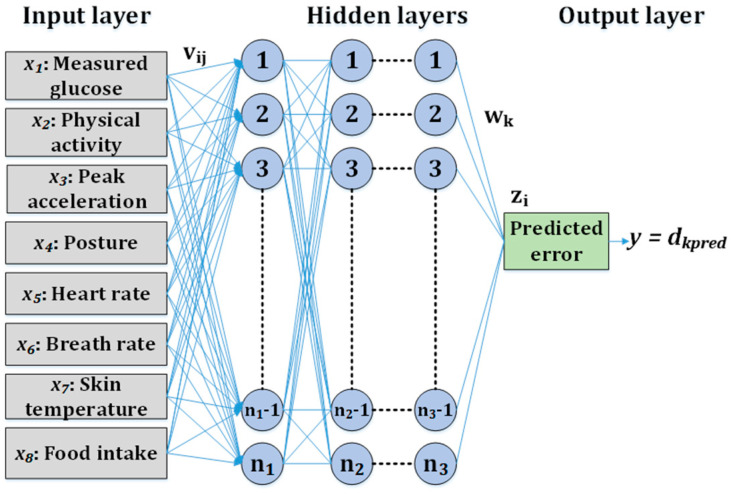
MLP-based CBGM regressor network diagram.

The network diagram for the MLP-based CBGM regressor is shown in Figure 3. Eight features were present in the input layer and one feature was present in the output layer, as shown in Figure 2. The input features were as follows: measured blood glucose values $\left(g_{m}\right.$ or $\left.x_{1}\right)$, physical activity $\left(x_{2}\right),$ peak acceleration $\left(x_{3}\right)$, posture $\left(x_{4}\right)$, heart rate $\left(x_{5}\right)$, breath rate $\left(x_{6}\right)$, skin temperature $\left(x_{7}\right)$, and food intake $\left(x_{8}\right)$. The output feature was the error prediction in glucose value ($d_{kpred}$), as shown in Figure 3. The predicted blood glucose values $\left(g_{p}\right)$ were calculated based on error prediction. The input and output layers were connected by several hidden layers. Each of the hidden layers had several perceptrons.

A network with one hidden layer with i perceptrons was considered to establish the mathematical model for MLP-based CBGM. The initial error in measured blood glucose values was calculated using Equation (1). During the forward pass, the output of each perceptron was calculated using Equation (2), and the predicted error in measured glucose values was calculated using Equation (3). During the backward pass, the error in the predicted error in the measured blood glucose value was calculated using Equation (4). A stochastic gradient descent (SGD) was used to calculate the error in the weight using Equations (5) and (6). Later, in the next iteration, the weights were updated using Equations (7) and (8). Finally, the predicted blood glucose values were calculated using Equations (9) and (10).

The number of hidden layers and the number of perceptrons in each hidden layer were decided using the grid search algorithm 1. We implemented a grid search for the MLP-based CBGM regressor. After a few trials, we shortlisted hidden layer (l), learning rate (α), learning type, activation function, solver type, and the number of iterations (t) as key parameters, which impacted the predicted error and hence the predicted blood glucose value.

Initial error:

(1) $$d_{k}(t)=x_{1}(t)-g_{r}(t)$$

Forward pass:

(2) $$z_{i}(t)=f\left(\sum \;v_{ij}(t)\times x_{j}(t)\right)$$

(3) $$y(t)=f\left(\sum \;w_{k}(t)\times z_{j}(t)\right)$$

Backward pass:

(4) $$E(t)=\frac{1}{2}\sum \;(d(t)-y(t){)}^{2}$$

(5) $$\frac{\partial E}{\partial w_{k}}=\left(d_{k}(t)-y(t)\right)\times \frac{\partial f\left(q_{k}(t)\right)}{f}\left(q_{k}(t)\right)\partial w_{k}$$

(6) $$\begin{array}{rr} & \frac{\partial E}{\partial v_{ij}}=f\left(P_{ij}(t)\times \sum \;\frac{\partial E}{\partial w_{k}}\times w_{k}(t)\right) \end{array}$$

Next iteration:

(7) $$w_{k}(t+1)=w_{k}(t)-\alpha \times \frac{\partial E}{\partial w_{k}}$$

(8) $$V_{ij}(t+1)=v_{ij}(t)-\alpha \times \frac{\partial E}{\partial v_{ij}}$$

Predicted blood glucose values:

(9) $$d_{kpred}(t)=y(t)$$

(10) $$g_{p}(t)=x_{1}(t)-d_{kpred}(t)$$

Due to the limitation of experimental paired data, smaller hidden layers with fewer perceptrons were used for our proposed approach for CBGM. We chose seven different options for hidden layers and perceptrons. These options included 20, 100, and 200 perceptrons for a single layer; 10 and 20 perceptrons each for two layers; and 10 and 20 perceptrons each for four layers. In addition, we chose a wide range of learning rates including 0.001, 0.01, 0.05, 0.1, 0.5, and 1. We considered three possible learning types available in the MLP regressor, i.e., constant, invscaling, and adaptive. As most of the input features were continuous data, we chose the tangent function (Tanh) and rectified linear unit (ReLu) as activation functions. We considered limited-memory Broyden–Fletcher–Goldfarb–Shanno (LBFGS), SGD, and adaptive moment estimation (ADAM) as solvers for the MLP-based CBGM regressor owing to their high performance in the field of neural networks. The simulation dataset had 166 sets of readings; hence, we considered 100, 500, and 1000 iteration steps sufficient for training. We chose momentum as 0.9 and 0.99 owing to the limited number of datasets. Grid search was nested as an optimizer, followed by momentum, number of iterations, activation function, learning rate (solver) type, learning rate, and hidden layers to build a robust model as represented in Algorithm 1. These options resulted in 4536 possible combinations for the MLP-based CBGM regressor.
**Algorithm 1** Grid search for hyperparameter optimization.HL: hidden layers
LRI: learning rate initial value
LRT: learning rate type
AF: activation function
MI: maximum iterations
Mom: momentum
Opt: optimizer
MLP: multilayer perceptron
D_g: dataset for group g (glucose range based)
1. Input: HL, LRI, LRT, AF, MI, Mom, Opt, MLP, D_g;
2. Initialize: Best_Model = null; model.MARD_temp=0, model.RMSE_temp=0;
3. for D_g in D:
4.  for HL_i in HL:
5.    for LRI_i in LRI:
6.      for LRT_i in LRT:
7.        for AF_i in AF:
8.          for MI_i in MI:
9.            for Mom_i in Mom:
10.              for Opt_i in Opt:
11.                 model = MLP(HL_i, LRI_i, LRT_i, AF_i, MI_i, Mom_i, Opt_i)
12.                    model.train(D_g_train)
13.                     model.test(D_g_test)
14.                     calculate model.MARD
15.                     calculate model.RMSE
16.                     model.MARD_temp{ } ← model.MARD
17.                  model.RMSE_temp{ }  ← model.RMSE
18.                     find (min(model.RMSE_temp)&&min(model.MARD_temp))
19.                  Best_Model(D_g) = model
20.  end
21.  Output: Best_Model


## 3. Result

The MLP-based CBGM approach was applied to the D1NAMO dataset accurately predict blood glucose values.

### 3.1. MLP-Based CBGM Grid Search Results

We executed several runs for the MLP-based CBGM regressor. Each time, 4536 combinations were checked for optimization.

Table 3 summarizes the list of optimized hyperparameters for each cluster. In the MLP-based CBGM software, the selection criteria were based on the smallest RMSE and MARD values. We also ensured that the maximum and minimum errors were reasonably reduced. The first column in Table 3 presents the cluster number, and the second column represents the blood glucose range. For cluster 0 (<80 mg/dL), the optimized hyperparameters were 2 hidden layers with 10 neurons in each layer, 0.1 as the learning rate, ReLu as the activation function, and the ADAM solver with 200 iterations. These optimized hyperparameters resulted in the smallest RMSE and MARD for cluster 0. Similarly, the optimized parameters for all other clusters, i.e., clusters 1–5, are listed in Table 3.

### 3.2. MLP-Based CBGM RMSE and MARD Calculations

Table 4 presents a summary of the RMSE, MARD, maximum error, and minimum error for each cluster. For cluster 0 (<80 mg/dL), the RMSE reduced from 19.6% to 9.6% and the MARD reduced drastically from 26.6% to 11.6%. The maximum and minimum errors reduced from 41.9% to 25% and −39.2% to −38.8%, respectively. The RMSE, MARD, and maximum error of the predicted blood glucose values were 40–50% lower than those of the measured values. A minor reduction in the minimum error was observed for cluster 0.

Similarly, Table 4 shows results for clusters 1–5. The RMSE and MARD were reduced for each of the clusters 1–5. The minimum and maximum errors were also reduced for most of the clusters.

The last row in Table 4 presents the overall result for the entire range of MLP-based CBGM in the D1NAMO dataset. The overall RMSE reduced from 30.3% to 13.3%. Our proposed concept proves the robustness of the prediction. The MARD was reduced significantly from 17.8% to 8.5% for the predicted blood glucose levels. The maximum and minimum errors were also reduced from 50% to 25% and from −120% to −38.8%, respectively. These results indicate that our proposed approach accurately predicted the error in measured blood glucose levels, adjusted the error, and provided accurately predicted blood glucose levels.

**Figure 4:**
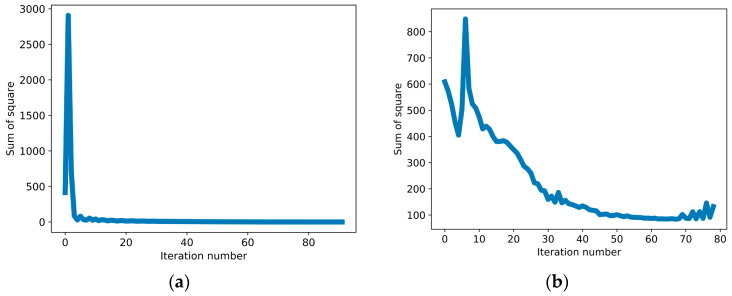

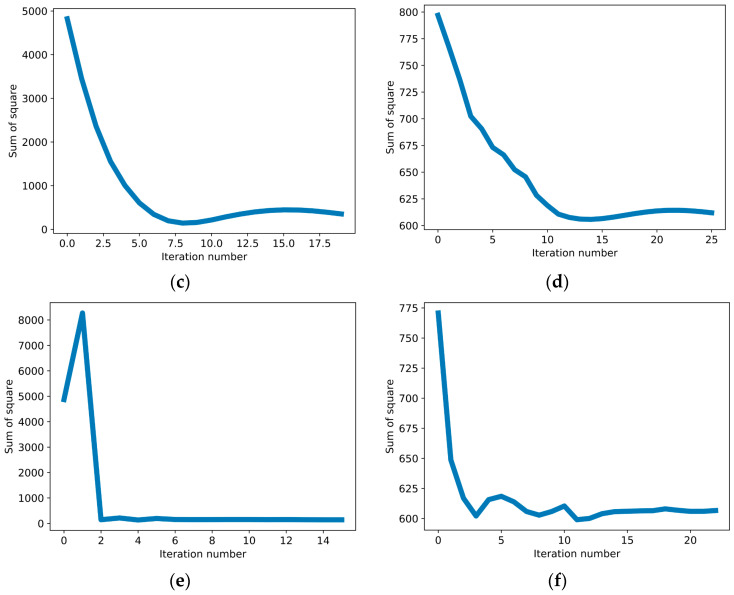
Sum of square error (SSE) plots for different clusters used to avoid underfitting and overfitting during hyperparameter optimization. (**a**) Cluster 0 (<80 mg/dL), the SSE converges to very low values within a few iterations after the initial increase. (**b**) Cluster 1 (81–115 mg/dL), the SSE reduces gradually, and by the 50th iteration, it reaches a low value. (**c**) Cluster 2 (116–150 mg/dL), the SSE reduces from the beginning and quickly converges. (**d**) Cluster 3 (151–180 mg/dL), the SSE converges within a few iterations. (**e**) Cluster 4 (181–250 mg/dL), the SSE converges within a few iterations after the initial spike. (**f**) Cluster 5 (>250 mg/dL), the SSE reduces consistently and converges from the 5th iteration onwards.

The training plots are shown in Figure 4, which were used to ensure robust hyperparameter optimization without underfitting or overfitting, as we had a limited number of input datasets. Figure 4a represents the SSE for cluster 0. The SSE reduced to a very low value within some iterations after an initial increase. Similarly, for cluster 1 (Figure 4b), the SSE reduced gradually and reached a low value by the 50th iteration. For cluster 2 (Figure 4c) and cluster 3 (Figure 4d), the SSE reduced from the beginning and quickly converged. For cluster 4, the SSE converged within a few iterations after the initial spike (Figure 4e). Finally, for cluster 5 (Figure 4f), the SSE reduced consistently and converged from the fifth iteration onwards. The SSE converged for all six clusters during training. This proves that our grid search for hyperparameter optimization worked well and was a good fit for training and prediction.

### 3.3. CEGA Plot

**Figure 5:**
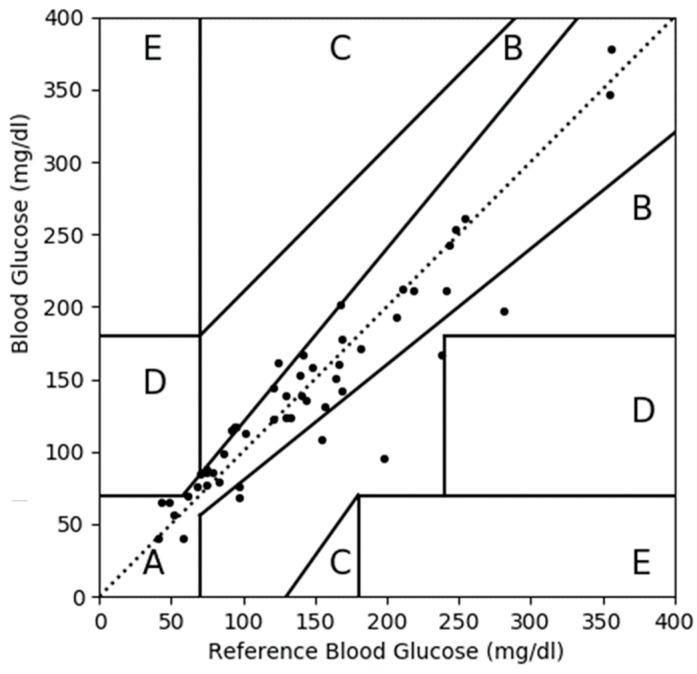
CEGA plot for the measured values with respect to reference values before applying MLP-based CBGM on the D1NAMO dataset. Paired data points are distributed with 39 (76%) in zone A, 11 (22%) in zone B, and 1 (2%) in zone D. No data points in zone C and E.

We also developed a CEGA plot for the measured blood glucose values versus the reference values obtained from the D1NAMO dataset, as shown in Figure 5. The plot showed that 51 paired data points were located in zones A, B, and D. Table 5 summarizes the CEGA plot for the measured values. Before applying our proposed approach, 39 (76%), 11 (22%), and 1 (2%) paired data points were located in zones A, B, and D, respectively.

**Figure 6:**
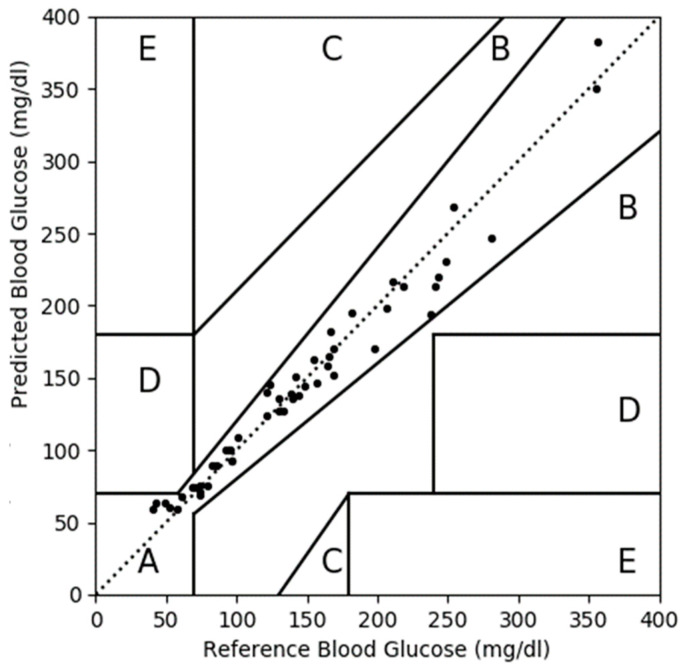
CEGA plot for the predicted blood glucose values with respect to reference values after applying the MLP-based CBGM approach on the D1NAMO dataset. All 51 (100%) paired data points were located in zone A. No data points in zone B to E.

After applying the MLP-based CBGM approach to the D1NAMO dataset, we developed a CEGA plot for the predicted blood glucose levels versus the reference values, as presented in Figure 6. All 51 (100%) paired data points were located in zone A, as shown in Figure 6. The CEGA plot for the predicted blood glucose after applying MLP-based CBGM is also summarized in Table 5. These results indicate that our proposed MLP-based CBGM algorithm accurately predicted blood glucose levels.

### 3.4. MLP-Based CBGM Error Plot

**Figure 7:**
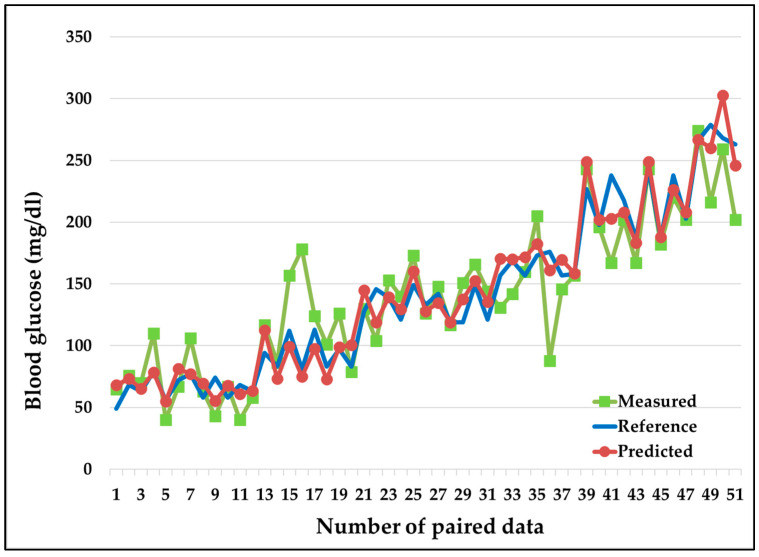
Performance of measured blood glucose (in green, before applying the MLP-based CBGM approach) and predicted blood glucose (in red, after applying the MLP-based CBGM approach) with respect to the reference invasive blood glucose (blue). Predicted values (red) following the reference values (green) owing to the significant reduction in errors.

Figure 7 shows that the initially measured values (in green) had significant errors with respect to the reference values. Later, after applying MLP-based CBGM, the predicted values (in red) were consistent with the reference values (in blue) owing to the accurate prediction of blood glucose.

## 4. Discussion

Several CBGM sensors and algorithms have been developed, but their accuracy still needs to be improved. The accuracy of CBGM depends on patients’ personalized parameters due to within-patient and between-patient variations.

Our proposed approach involved clustering and training different machine learning models for each cluster based on personalized patient data, such as patients’ physical activities, posture, heart rate, breath rate, skin temperature, and food intake. This approach can possibly improve the accuracy of CBGM. Using these personalized parameters, an improved prediction model was developed to accurately predict blood glucose levels for each knowledge-based cluster for CBGM.

We successfully executed our approach on the D1NAMO dataset. The MLP-based CBGM approach outperformed all other machine learning models. Each cluster was trained separately, and the hyperparameters were independently optimized using a grid search to achieve higher prediction accuracy. After applying the MLP-based CBGM approach to the D1NAMO dataset, the MARD reduced from 17.8% to 8.5%. The CEGA plot showed improvement; all paired data points for the predicted blood glucose values were located in zone A compared with 76% of the data points in zone A, 22% in zone B, and 2% in zone D for the measured values. The maximum and minimum errors were reduced from 50% to 25% and −120% to −38.8%, respectively. In the present study, the proposed approach was applied to a limited size of the D1NAMO dataset. We will further determine the accuracy of this approach after applying it to different and larger datasets in the future.

## 5. Conclusions

As glucose concentration in the blood is <1% of the total blood volume, it is difficult to accurately measure the blood glucose level using CBGM sensors that use interstitial fluid as a biological medium for measurement. Blood glucose levels greatly vary based on patients’ personalized parameters, as they influence within-patient and between-patient variations, making continuous blood glucose monitoring even more challenging. To increase the accuracy of CBGM and effectively handle errors, we scientifically divided the blood glucose range into six knowledge-based clusters and trained each cluster using machine learning models and patients’ personalized data, such as physical activity, posture, heart rate, breathing rate, skin temperature, and food intake. The selected and trained machine learning models accurately predicted values for CBGM. The proposed approach was successfully applied to the D1NAMO dataset, which resulted in an improvement in the MARD from 17.8% to 8.5% for MLP-based CBGM, and all data points were located in zone A of the CEGA plot for the predicted blood glucose. We plan to apply the proposed approach to different and larger datasets.

## 6. Patents

The patent “Personalized blood glucose measurement device using machine learning technique” was filed in US PTO.

## Figures and Tables

**Table 1:** List of symbols.

Symbol	Unit	Definition
*g_r_*	mg/dL	Reference invasive blood glucose values
*d_k_*	mg/dL	Error in measured CBGM values
*g_m_* or *x_1_*	mg/dL	Measured CBGM values
*x_2_*	g	Physical activity
*x_3_*	g	Peak acceleration
*x_4_*	Degrees	Body posture
*x_5_*	BPM	Heart rate
*x_6_*	BPM	Breath rate
*x_7_*	°C	Skin temperature
*x_8_*	Calories	Food intake
*y or* *d_kpred_*	mg/dL	Predicted error in CBGM values
*g_p_*	mg/dL	Predicted CBGM value
*X_j_*	mg/dL	Set of data for *x_j_*
*Y*	mg/dL	Set of data for *y*
*G_r_*	mg/dL	Set of data for *g_r_*
*G_m_*	mg/dL	Set of data for *g_m_*
*G_p_*	mg/dL	Set of data for *g_p_*
*E_1_*	%	Initial MARD before applying MLP
*E_2_*	%	Final MARD after applying MLP
*v_ij_*		First hidden layer weights for MLP
*w_k_*		Output layer weights for MLP
*f(p_ij_(t))*		Activation function in the hidden layer
*f(q_k_(t))*		Activation function in the output layer
*E(t)*		Sum of square error
*z_i_(t)*		Output of hidden layer
*l*		Number of hidden layers
*i*		Number of perceptrons in each hidden layer
*j*		Number of independent variables (input)
*α*		Learning rate
*m*		Training momentum
*t*		Number of iterations

**Table 2:** Initial results of the CBGM machine learning model.

ML Model	MARD	CEGA Plot Zone (%)				
		A	B	C	D	E
SVR	24.9	55	36	0	9	0
KNN	23.9	60	32	0	9	0
DT	17.4	70	26	0	4	0
RF	16.6	74	19	0	6	0
AdaBoost	15.6	79	17	0	4	0
MLP	14.4	83	15	0	2	0

**Table 3:** Optimized hyperparameters for the MLP-based CBGM regressor.

#	Range	Optimized Hyperparameters
0	<80	l = 2, i = 10, α = 0.1, adaptive, ReLu, ADAM, t = 200, m = 0.99
1	81–115	l = 4, i = 10, α = 0.1, adaptive, ReLu, ADAM, t = 500, m = 0.9
2	116–150	l = 4, i = 20, α = 0.001, adaptive, ReLu, ADAM, t = 500, m = 0.99
3	151–180	l = 1, i = 20, α = 0.1, invscaling, ReLu, ADAM, t = 1000, m = 0.9
4	181–250	l = 4, i = 20, α = 0.1, constant, ReLu, ADAM, t = 1000, m = 0.99
5	>250	l = 1, i = 100, α = 0.1, adaptive, Tanh, ADAM, t = 200, m = 0.99

# represents the cluster number for each blood glucose range. MLP-based CBGM regressor optimizes hyperparameters separately for each cluster for accurate prediction of blood glucose.

**Table 4:** Test results of the MLP-based CBGM regressor.

#	RMSE		MARD (%)		Max Error (%)		Min Error (%)	
	Initial	Final	Initial	Final	Initial	Final	Initial	Final
0	19.0	9.6	23.6	11.6	41.9	25.0	−39.2	−38.8
1	40.7	12.6	31.8	12.4	4.8	13.7	−120	−21.0
2	21.0	13.1	12.8	7.8	28.8	18.6	−26.9	−15.6
3	38.4	11.2	15.8	5.9	50.0	8.4	−18.5	−9.4
4	26.5	15.2	7.4	4.9	29.8	14.8	−7.0	−9.8
5	44.3	21.7	13.0	6.7	23.2	6.9	−3.0	−13.0
All	30.3	13.3	17.8	8.5	50.0	25.0	−120	−38.8

# represents the cluster number for each blood glucose range. MLP-based CBGM optimized hyperparameters based on the smallest final RMSE, smallest final MARD, smallest final maximum (Max) error, and smallest final minimum (Min) error. The last row is a summary of the overall (entire range) performance of the MLP-based CBGM approach.

**Table 5:** CEGA plot summary.

Zone	Before ^1^		After ^2^	
	Number	%	Number	%
A	39	76	51	100
B	11	22	0	0
C	0	0	0	0
D	1	2	0	0
E	0	0	0	0

^1^ Measured values with respect to reference values before applying the MLP-based CBGM approach on the D1NAMO dataset. ^2^ Predicted values with respect to reference values after applying the MLP-based CBGM approach on the D1NAMO dataset.

## Data Availability

The data are not publicly available as patent application pending.
